# Supplementary material for: Thromboelastometry demonstrates endogenous coagulation activation in nonsevere and severe COVID-19 patients and has applicability as a decision algorithm for intervention
Source: PLoS One. 2022 Jan 14;17(1):e0262600. doi: 10.1371/journal.pone.0262600 (PMC8759688; doi:10.1371/journal.pone.0262600)
Supplement: S1 Table — EXTEM = Extrinsic coagulation activity assay; INTEM = Intrinsic coagulation activity assay; NATEM = Non-Activated coagulation assay and FIBTEM = functional assessment of Fibrinogen assay. CT (expressed in seconds) = clotting time: refers to thrombin formation, timeframe from activation until an amplitude of 2mm; ALPHA (expressed in °) = defined as the angle between the middle axis and the tangent to the clotting curve through the 2mm amplitude point; CFT (expressed in seconds) = clot formation time: refers to the dynamic formation of fibrin, timeframe between 2 mm and 20 mm of clot amplitude; MCF (expressed in mm) = maximum clot firmness: refers to the maximum firmness of the clot, proportional to the amount of fibrinogen and platelets; ML (expressed in %) = maximum lysis: represents the percentage of clot reduction after initiation of fibrinolysis. § Data are expressed as Mean±SD. † P values were calculated with independent-samples Student’s t-tests for continuous variables. Significant differences are underscored by letters “a”, “b” and “c” for pairwise comparisons between NS vs HC, S vs HC and S vs NS, respectively. (DOCX) [file pone.0262600.s001.docx]

**Supplementary Table 1. Thromboelastometry Parameters in COVID-19 Patients and Healthy Controls§**

|  | **Healthy Control (HC, n=09)** | **Patients (COVID, n=41)** | **P value†** | **Non-severe (NS, n=20** | **Severe (S, n=21** | **P value †** |
| --- | --- | --- | --- | --- | --- | --- |
| EXTEM |  |  |  |  |  |  |
| CT | 56±7 | 65±10 | 0.02 | 61±9 | 68±9^b,c^ | 0.002^b^/0.02^c^ |
| ALPHA | 75±4 | 77±5 | NS | 74±5 | 79±2^b,c^ | 0.0006^b^/0.0007^c^ |
| CFT | 83±24 | 69±28 | NS | 81±33 | 57±14^b,c^ | 0,0009^b^/0.004^c^ |
| MCF | 65±5 | 68±6 | NS | 65±5 | 70±5^b,c^ | 0.007^b^/0.001^c^ |
| ML | 6±3 | 6±3 | NS | 7±3 | 6±2 | NS |
| TPI | 3±2 | 4±2 | NS | 3±2 | 4.8±2^b,c^ | 0.009^b^/0.003^c^ |
| INTEM |  |  |  |  |  |  |
| CT | 189±25 | 176±30 | NS | 181±30 | 171±29 | NS |
| ALPHA | 77±3 | 78±3 | NS | 76±3 | 79±2^b,c^ | 0.009^b^/0.003^c^ |
| CFT | 67±17 | 60±17 | NS | 68±19 | 52±10^b,c^ | 0.009^b^/0.003^c^ |
| MCF | 63±6 | 67±6 | NS | 63±5 | 69±5^b,c^ | 0.006^b^/0.0007^c^ |
| ML | 7±3 | 9±4 | NS | 9±3 | 8±3 | NS |
| TPI | 3±1 | 4±2 | NS | 3±1 | 5±2^b,c^ | 0.01^b^/0.001^c^ |
| NATEM |  |  |  |  |  |  |
| CT | 417±211 | 283±108 | 0.008 | 269±115^a^ | 284±120^b^ | 0.02^a^/0.03^b^ |
| ALPHA | 66±8 | 72±8 | 0.01 | 69±9 | 75±5^b,c^ | 0.0009^b^/0.007^c^ |
| CFT | 124±50 | 123±95 | NS | 122±94 | 73±31 | 0.002^b^/0.03^c^ |
| MCF | 61±6 | 67±6 | 0.008 | 63±5 | 69±4^b,c^ | 0.0002^b^/0.0005^c^ |
| ML | 6±4 | 7±4 | NS | 7±4 | 6±3 | NS |
| TPI | 2±1 | 3±2 | 0.01 | 2±1 | 4±2^b,c^ | 0.0005^b^/0.0003^c^ |
| FIBTEM |  |  |  |  |  |  |
| MCF | 19±4 | 27±9 | 0.01 | 20±6 | 33±7^b,c^ | 0.0001^b^/0.0001^c^ |

EXTEM = Extrinsic coagulation activity assay; INTEM = Intrinsic coagulation activity assay; NATEM = Non-Activated coagulation assay and FIBTEM = functional assessment of Fibrinogen assay. CT (expressed in seconds) = clotting time: refers to thrombin formation, timeframe from activation until an amplitude of 2mm; ALPHA (expressed in ^o^) = defined as the angle between the middle axis and the tangent to the clotting curve through the 2mm amplitude point; CFT (expressed in seconds) = clot formation time: refers to the dynamic formation of fibrin, timeframe between 2 mm and 20 mm of clot amplitude; MCF (expressed in mm) = maximum clot firmness: refers to the maximum firmness of the clot, proportional to the amount of fibrinogen and platelets; ML (expressed in %) = maximum lysis: represents the percentage of clot reduction after initiation of fibrinolysis. § Data are expressed as Mean±SD. † P values were calculated with independent-samples Student’s t-tests for continuous variables. Significant differences are underscored by letters “a”, “b” and “c” for pairwise comparisons between NS vs HC, S vs HC and S vs NS, respectively.
