# Supplementary material for: Thromboelastometry demonstrates endogenous coagulation activation in nonsevere and severe COVID-19 patients and has applicability as a decision algorithm for intervention
Source: PLoS One. 2022 Jan 14;17(1):e0262600. doi: 10.1371/journal.pone.0262600 (PMC8759688; doi:10.1371/journal.pone.0262600)
Supplement: Attachment — Submitted filename: Response to Reviewers.docx [file pone.0262600.s002.docx]

Dear editors

This document is intended to provide responses to each point addressed by the Journal and Reviewers.

The authors would like to thank for all the suggestions made, including the inclusion of our protocol in the protocol repository: protocols.io (dx.doi.org/10.17504/protocols.io.bwvbpe2n)

**Journal Requirements**

1. Re-checked PLOS ONE´s style requirements.

We found some situations where the Journal´s style requirements were not met. All of those were corrected.

2.	We decided to avoid Written Consent as the risk of manipulating objects between healthy and sick individuals could, theoretically, increase the viral transmission in the institution.

So, the use of digital consent was requested and approved by our ethic review board, namely CONEP (Comissão Nacional de Ética em Pesquisa).

Both informations were included in the manuscript, in the Methods section.

3. Amended the information included in the Funding Statement so it is the same mentioned in the Financial Disclosure.

4. The new text with Funding Statement was included at the end of the Cover Letter, as requested.

5. To a complete understanding of the results and conclusions made in the paper, all data were made available: not only in graphic visualization, but also in a Supporting Table with all averages, standard deviation and p values.

As suggested, we also uploaded all raw data to a public repository, namely Harvard Dataverse: https://doi.org/10.7910/DVN/NWSVZA

6. All authors were included in the Manuscript Submission Data.

**Reviewer responses to Questions**

1. Reviewer #1 answered positively; nothing to change.

2. Our statistical analysis was planned by three senior authors. Author MSG and LRA are both mathematicians at UFU (Federal University of Uberlandia) and actually work with the development of machine learning algorithms. Besides them, OAMF is a senior researcher with great expertise in bio-statistics and mathematic models of generalizability. They have been working together planning and participating in many studies involving the necessity of advanced mathematic evaluations. After that, other two authors, CPA and OTN, two researchers specially involved with statistical analysis, validated and re-tested all the data obtained in this study.

Besides that, all our raw data and statistical analysis are available in the public repository Harvard Dataverse (DOI included in the Supporting information): https://doi.org/10.7910/DVN/NWSVZA

3. All our raw data are public and there isn´t any objection to make it available. For the understanding of the results and conclusions made in the paper, the data included are enough, as we included not only the p value, but also, means and standard deviation. We understand that it may be more difficult to visualize the numbers just in the graphics, but in the S1 Table, all the numbers are available and may easily be accessed and analyzed.

If it is not enough, it will be a pleasure for us to provide access to all raw data (Public repository Harvard Dataverse): https://doi.org/10.7910/DVN/NWSVZA

4. Reviewer #1 answered positively; nothing to change.

5.

Thank you very much for the suggestion.

At the time of the research we were faced with the necessity of stratifying our patients. At that time, a used guideline available was published by the WHO and separated individuals using the criteria selected in this study. As the time passed, possibily we would use other criteria, but at that time, it was a reasonable one.

Besides the novel use of NATEM in general COVID patients, other important topic in our study was the finding that even nonsevere patients already have hemostatic alterations, especially in NATEM. Moreover, the original description of TPI (thrombodynamic index) is other strong fact in our study. This parameter, as NATEM CT, also maintained the alterations described even when not separating the COVID-19 patients.

Thinking in this perspective, even with more strict criteria it probably wouldn’t modify the results, as both groups, nonsevere and severe, showed endogenous activation, represented by shorter CT in NATEM. Moreover, TPI probably wouldn´t also be altered by more strict criteria for classifying patients as severe.

We respectfully consider that even changing the classification criteria, our findings justify the publication and highlight more aspects of this challenging disease. After all, we have commented in the discussion that our sample size wouldn’t be able to detect different severities in the severe group.

The suggestion of adding platelet count is very good also; unfortunately, it was not available in most of our patients. As these patients are from public service hospitals, even simple exams may be unavailable at some periods. That was the case during the inclusion period: platelets, d-dimers and even creatinine were unavailable and so, not considered for the mathematic models.

6. Reviewer #1 answered positively; nothing to change.

We hope that our answers are appropriate, but we are completely available to explain any other detail or doubt that may appear.
